# Prospective sampling bias in COVID-19 recruitment methods: experimental evidence from a national randomized survey testing recruitment materials

**DOI:** 10.1186/s12874-022-01726-2

**Published:** 2022-09-26

**Authors:** Eric B. Kennedy, Mia Charifson, Megan Jehn, Eric A. Jensen, Jenna Vikse

**Affiliations:** 1 Disaster and Emergency Management, School of Administrative Studies, York University, Toronto, Canada; 2 New York University School of Medicine, New York, USA; 3 Global Health, School of Human Evolution and Social Change, Arizona State University, Tempe, USA; 4 Research Department, Institute for Methods Innovation, Dublin, Ireland; 5 Discourse, Science, Publics Lab, Department of Psychology, University of Guelph, Guelph, Canada

**Keywords:** COVID-19, Survey research, Sampling bias, Response bias, Recruitment methods

## Abstract

**Background:**

In the context of the COVID-19 pandemic, social science research has required recruiting many prospective participants. Many researchers have explicitly taken advantage of widespread public interest in COVID-19 to advertise their studies. Leveraging this interest, however, risks creating unrepresentative samples due to differential interest in the topic. In this study, we investigate the design of survey recruitment materials with respect to the views of resultant participants.

**Methods:**

Within a pan-Canadian survey (stratified random mail sampling, *n* = 1969), the design of recruitment invitations to prospective respondents was experimentally varied, with some prospective respondents receiving COVID-specific recruitment messages and others receiving more general recruitment messages (described as research about health and health policy). All respondents participated, however, in the same survey, allowing comparison of both demographic and attitudinal features between these groups.

**Results:**

Respondents recruited via COVID-19 specific postcards were more likely to agree that COVID-19 is serious and believe that they were likely to contract COVID-19 compared to non-COVID respondents (odds = 0.71, *p* = 0.04; odds = 0.74, *p* = 0.03 respectively; comparing health to COVID-19 framed respondents). COVID-19 specific respondents were more likely to disagree that the COVID-19 threat was exaggerated compared to the non-COVID survey respondents (odds = 1.44, *p* = 0.02).

**Conclusions:**

COVID-19 recruitment framing garnered a higher response rate, as well as a sample with greater concern about coronavirus risks and impacts than respondents who received more neutrally framed recruitment materials.

**Supplementary Information:**

The online version contains supplementary material available at 10.1186/s12874-022-01726-2.

## Background

The COVID-19 crisis has led to a wave of survey-based research around the world, albeit sometimes of suspect quality [1]. Well-designed survey research in COVID-19 can help identify social impacts, measure attitudes, and document the ways respondents are adapting [2], which is critical to understanding public behaviors during the pandemic [3, 4]. Moreover, carefully designed social research can help to inform policy and response design [5] through providing real-time evidence about on-the-ground conditions and the effectiveness of various interventions.

The value of such research, however, can be seriously limited by methodological errors and biases [6, 7]. Investigations into COVID-19 survey research has already demonstrated the influence, for example, of biases introduced at the level of item design (e.g., how questions or prompts are formulated), such as the way that social desirability bias and research desirability bias can affect the responses that respondents offer in ways that undermine data quality regarding public behaviors [8–10]. Surveys can also be subject to systematic biases in who participates in the research, such as the influence of selection and non-response biases in amplifying the participation of certain groups over others [11, 12] or survivorship bias in cohort studies [13]. If particular groups that share a sociodemographic identity, for instance, are less likely to participate, the representativeness of the results could be compromised in ways that are difficult to perfectly control for later on [14].

In this paper, we look at an additional potential source of systematic bias: sampling bias induced by the specific recruitment instruments used for an online survey. The representativeness of even well-designed probability samples hinges on which prospective respondents actually participate rather than ignoring recruitment efforts, declining participation, or dropping out during the study. Previous research from the field of political science has suggested that recruitment messages can influence survey sample representativeness in political issue polling [15]. Equivalent research has not been conducted in the public health context or emergency context, however, to understand how these effects could play out during pressing health crises such as COVID-19. In this study, we aim to do this by using a national Canadian survey to investigate whether recruitment invitations introduce sampling bias.

In this paper, we compare the recruitment instruments (postcards advertising the survey) received by prospective respondents. We contrasted postcards that advertised the research as about “COVID” with others advertising a general health survey (see below for further details, and the supplementary materials for the exact postcard designs). We tested two hypotheses:

- H1. COVID-specific postcards will receive a higher response rate.
- H2. Respondents from the COVID-specific postcards will be more concerned about the coronavirus.

## Methods

During a national survey on COVID-19 in Canada [16], participants were recruited using a postcard-drive-to-web approach (i.e., households received physical postcards requesting that they complete an online survey, with both a URL and scannable QR code available). The sampling frame included all Canadian households with a mailing address. A random sample of 154,758 households was selected based on mail delivery routes, stratified by the urban/rural and apartment/house dwelling breakdown of Canada, while oversampling smaller provinces and territories.^1^ This sampling frame, obtained through a partnership with Canada Post, allowed for complete coverage of all Canadians with a mailing address. Canada Post conducted the randomized selection of mail delivery routes following instructions regarding these parameters by the research team.

Beginning March 23rd, 2020, prospective participants were sent a postcard requesting online survey participation, with a prize draw offered ($200 prizes). Postcards requesting respondents’ views on COVID-19 were sent to two-thirds of the sample, while the rest were asked for their views on ‘healthcare in Canada.’^2^ Postcard treatment groups (COVID vs. non-COVID) were randomized, again following the same stratifications mentioned above. Both postcard designs were consistent (bilingual, including both university and funder logos), varying only wording and image used, and can be reviewed as Figs. SM.1 and SM.2 in the supplementary material. For the purpose of this study, we only considered responses within the initial three-week period post-delivery (a small number of respondents completed the survey through the following weeks; they were excluded from analysis to minimize the influence of these temporarily long-tail responses, as case counts, government measures, and public perceptions changed rapidly throughout the crisis). Data were collected for three weeks (until April 12th, 2020) using the Qualia Analytics online survey platform, then deduplicated to ensure one response per person (retaining the most complete response during the survey period).

We examined four Likert-type items (options “Strongly Disagree”, “Disagree”, “Neutral”, “Agree” and “Strongly Agree”): (1) “Getting sick with COVID-19 can be serious” (2) “COVID-19 will NOT affect many Canadians” (3) “I will probably get COVID-19,” and (4) “The threat posed by COVID-19 is exaggerated by the Canadian federal government.”^3^ We used both polynomial ordinal logistic regression and Kruskal-Wallis chi-squared tests to compare responses from COVID and non-COVID recruitment types. COVID-19 specific respondents were the reference group for postcard type for polynomial ordinal logistic regressions. The proportional odds assumption of the ordinal regression was tested and did not find evidence to reject the assumption based off the data (*p* < 0.05) [17]. All analyses used R (version 4.0.2) using tidyverse, readr, ggpubr, ggplot2, HH, MASS, and lsr packages (code and raw data available upon request; see data availability statement).

To control for potential demographic variation between respondents from the two postcard types, we tested for age, gender, level of education and racial identification as possible covariates through t-tests or chi-squared test (*p*-value threshold of *p* < 0.05). Significant covariates (age, gender, and region) were identified and included in all analyses. Given that data collection spanned a three-week period, we also include week of response and region^4^ as covariates to help account for the changing epidemiological, political, and social landscape.^5^

## Results

A total of *n* = 1969 participants responded during the initial two-week window and passed data cleaning/verifications. The average age of the sample was 49 years old (SD = 16.73), but differed by respondent group, with the general health group being slightly older (B = 3.91, *p* = 0.001) (Supplementary Table SM.2). Regional distribution of respondents also differed between the two respondent groups (χ^2^ = 16.95, Cramer’s V = 0.09, *p* = 0.005), with respondents from Ontario and Quebec (the two most populous provinces) slightly more heavily represented within the COVID postcards (Supplementary Table SM.2). That is, if postcard type was known, 9% of the variability in respondent location (by province) could be accurately predicted (and vice versa). Gender also varied (χ^2^ = 4.44, Cramer’s V = 0.05, *p* = 0.04) with a slightly higher proportion of female respondents in the general health respondent group (Supplementary Table SM.2). That is, if postcard type was known, then 5% of the variability in gender could be accurately predicted (and vice versa). There was no statistically significant difference in racial identification or level of education between postcard categories (Supplementary Table SM.2).

- H1. COVID postcards would receive a higher response rate than health postcards.

Confirming the hypothesis, there was a marked difference in response rates between the two postcards. Despite mailing 50,082 general health postcards, only 243 responses came from this recruitment instrument (a response rate of only 0.49%). By contrast, there were 1730 respondents from the pool of 104,676 COVID-19 postcards, or a response rate of 1.65%. This suggests topical postcards can be used to return a much higher response rate than more ‘neutral’ recruitment messages, especially in the context of a public health emergency generating significant public attention (as was the case particularly in early 2020, prior to potential respondent fatigue from oversaturation of COVID-19 surveys).

- H2. Respondents from the COVID postcards demonstrated a higher degree of concern about the coronavirus than the health sample.

**Fig. 1:**
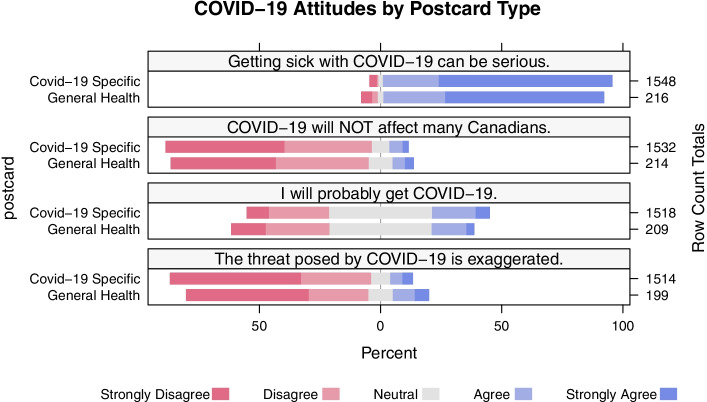
Differences in COVID-19 risk perceptions by recruitment type (COVID-specific vs. general health)

This increased response rate, however, comes with a tradeoff: even controlling for variation in age, gender, and regional distribution, there was variation between the perspectives expressed by respondents from each postcard. We examined four COVID risk perception questions (see Fig. 1), finding three with statistically significant variation.

The overall response distribution differed between the two respondent groups (K-W χ^2^ = 4.09, *p* = 0.04) when responding to the statement “Getting sick with COVID-19 can be serious”. Adjusting for age, gender, region and week of response, non-COVID postcard respondents were less likely to agree with this statement than COVID-19 specific postcard respondents (adjusted odds ratio (aOR) = 0.714, 95% CI = 0.522–0.976) (Supplementary Table SM.3). Similarly, for the statement “I will probably get COVID-19” respondent groups’ views diverged (K-W χ^2^ = 6.81, *p* = 0.001) with non-COVID postcard respondents less likely to agree with the statement (aOR = 0.739, 95% CI = 0.561–972) (Supplementary Table SM.4). Consistent with these findings, generic health postcard respondents were more likely to agree that “the threat posed by COVID-19 is exaggerated” than COVID-19 specific respondents (aOR = 1.441, 95% CI = 1.073–1.935), again revealing a statistically significant difference between the two respondent groups (K-W χ^2^ = 2.85, *p* = 0.09) (Supplementary Table SM.5). There was no statistically significant variation between the groups in responses to the statement “COVID-19 will not affect many Canadians” (K-W χ^2^ = 2.60, *p* = 0.11; aOR = 1.213, 95% CI = 0.918–1.604) (Supplementary Table SM.5).

## Discussion

Surveys can be an invaluable tool for collecting public opinion and experiences on emerging crises like COVID-19. They are vulnerable, however, to biases that can arise thanks to problems in the methodological design of the study. Our study advances this literature by identifying and documenting a subtle manifestation of bias in the context of COVID-19 research; namely, the way that recruitment materials themselves can shape who elects to respond and/or how they elect to participate.

While this experiment documents the results of this bias, there are multiple possible interpretations of the mechanisms by which it emerges. A straightforward possibility is that the COVID-19 postcards created a sampling bias, wherein the specificity of this topic – and massive public attention – more strongly motivates participation from those who hold more concerned attitudes (as opposed to, say, someone uninterested discarding the postcard). Alternative possibilities, however, may also be present. For example, researcher demand bias (i.e., respondents perceiving and seeking to fulfil what they believe the researcher hopes to hear; in this case the postcard suggesting researchers who had concerns about COVID-19) or priming (i.e., an initial stimulus that affects primacy of particular topics in later responses; in this case the postcard subject matter making salient COVID-19) are both possible alternative explanatory frameworks. A reviewer also pointed out the possible role of ‘Malmquist bias’ [18], given a more appealing image on the COVID postcards (graphical representation of the virus versus a drab operating theatre). Further research could help to differentiate between these causal mechanisms in experimental conditions.

For public health practitioners and researchers, these findings have several practical implications. While sampling bias is often thought of in ‘obvious’ examples (e.g., missing key demographics in recruitment), our findings illustrate that significant biases can occur in subtler ways, like the design of recruitment messages. As such, it is critical that researchers think carefully about – and test – recruitment tools they use and transparently share the tools they used for reviewer and reader examination. Likewise, practitioners should critically assess survey recruitment strategies before relying on the findings, lest sampling bias lead to unrepresentative findings. These lessons are critical in the context of the COVID-19 pandemic, as a large portion of survey research has explicitly used COVID-19 recruitment messages as a way of garnering higher rates of public participation – while potentially introducing the biases we’ve identified here.

We also find that targeted messages can be useful for increasing response rate. However, these increased response rates are hindered by the potential of over-sampling those with higher levels of concern. As such, researchers should consider using recruitment instruments that use more generic framings to minimize risks of sampling bias, or analytic strategies to calibrate for context-specific skews.

There are, of course, limitations to this study. For example, the response rate of both recruitment methods was remarkably low. While this is not uncommon in mail-based recruitment, other studies during the pandemic using similar methods have achieved higher response rates [19, 20]. Further investigation could be done to isolate possible COVID-specific effects (e.g., early concerns about fear of transmission on the surface of mail), the particulars of this study (e.g., whether aspects like the size, material, or design of the postcards affected outcomes), or mail solicitation in general. Moreover, the ‘health’ framing does not represent a true ‘neutral’ option: while it was certainly a more generic recruitment than a COVID-specific advertisement, it likely comes with its own biases as compared to other possible recruitment materials. As discussed above, it is also very difficult to come up with a theoretically justifiable method for controlling for ‘local risk,’ a highly subjective variable. More work should be done on this topic to develop techniques to account for these perceptions. Finally, as a reviewer helpfully pointed out, there are several other correlates and variables – such as anxiety, depression, and physical health – that would be very interesting to explore to understand their potential impacts on response bias. These are important topics, albeit highly complex ones (e.g., understanding the relationship between pre-existing mental health conditions, COVID-induced or exacerbated ones, and response biases) which warrant a fuller investigation in future research.

## Conclusion

Here, we found that using recruitment invitations that explicitly reference COVID-19 increased participation but also increased respondent degrees of concern about the health crisis. A likely explanatory pathway is the presence of sampling bias, wherein the recruitment instrument affected which potential respondents were likely to actually participate. Researchers and consumers of research should be especially careful in situations like COVID-19, wherein there is a tendency to explicitly use a topic of great public importance (especially in context of already problematic methodologies, like convenience sampling) as a way of increasing response rates. Recruitment messages that foreground hot-button issues, like COVID-19, can inadvertently skew their own results through systematic biases.

## Supplementary Information

**Additional file 1: Table SM.1.** Expected (based on population) versus actual count of households receiving postcards. **Fig. SM.1.** “COVID Specific” postcard design. **Fig. SM.2.** “General Health” postcard design. **Table SM.2.** Descriptive characteristics of study sample by postcard type. **Table SM.3.** Likelihood of Agreeing with the statement: “Getting sick with COVID-19 can be serious.” **Table SM.4.** Likelihood of Agreeing with the statement: “I will probably get COVID-19.” **Table SM.5.** Likelihood of Agreeing with the statement: “The threat posed by COVID-19 is exaggerated by the Canadian federal government.” **Table SM.6.** Likelihood of Agreeing with the statement: “COVID-19 will NOT affect many Canadians.”.

## Data Availability

The datasets and analysis files used in the article are available from the corresponding author upon reasonable request. The entire survey dataset will be made available open-access at the conclusion of the study; see https://www.cemppr.org/research/covid-19-in-canada for more details and updates when released.

## Footnotes

1 For a detailed breakdown of the oversampling, please see Table SM.1. in the Supplementary Materials, which demonstrates the expected number of households sampled in each province vs. the actual number sampled, as well as a percentage of expected (> 1 represents oversampling; < 1 represents under-sampling).

2 The uneven split reflects a compromise between this particular investigation and the overall objectives of the survey project (which tracks attitudes on a wide variety of topics). At the time – and continuing today – the default for COVID-specific online surveys is to use COVID-specific recruitment messages. Moreover, while Hypothesis 1 (improve response rate for COVID-specific postcards) seemed highly likely (and therefore worth leveraging), not all members of the overall research consortium running the survey were persuaded by Hypothesis 2 (that, in the context of March 2020 with heightened interest in the topic across the ideological spectrum, it would result in systematic bias in who responded). As such, this compromise position (a one-third/two-third split) allowed the opportunity to investigate this default practice, inform calibration of our own work if such a bias did exist, and provide a higher response rate for those persuaded by the first – but not the second – hypothesis.

3 For observers of Canadian healthcare, which is generally a provincial responsibility (although local health agencies/officers and federal counterparts have played a very active role, given the all-encompassing nature of COVID), we selected *federal* government for this question for two reasons. First, in early 2020, when the survey was designed, the federal government played an outsized role in managing the crisis (e.g., in discussions about border closures; in the role of the Public Health Agency of Canada in disease monitoring and initial response). Second, because the question was asked of respondents from across the country, asking their perception of *federal* rhetoric helped to eliminate varying provincial responses/rhetoric as a complication. In Kennedy et al. 2020 and other work, we explore other items from the survey designed to specifically investigate federal, provincial, and local differentiation (e.g., trust in different levels of government; information sources; accepted policies; etc).

4 Regions used were British Columbia, Ontario, Quebec, Prairies, Maritimes, and Territories.

5 Selecting the appropriate controls for ‘local’ case counts is a fraught issue. It is not clear what participants would define as ‘local’ (e.g., within city, region, province; nearby home vs. work vs. loved ones; etc). While we use the imperfect proxies of date of response and region in this paper to serve as a check, further work should be done on what representation of risk have been most salient to members of the public (e.g., are members of the public considering provincial caseloads, local caseloads, caseloads in the area they work or that parents live, etc).
