# Supplementary material for: Prospective sampling bias in COVID-19 recruitment methods: experimental evidence from a national randomized survey testing recruitment materials
Source: BMC Med Res Methodol. 2022 Sep 26;22:251. doi: 10.1186/s12874-022-01726-2 (PMC9510455; doi:10.1186/s12874-022-01726-2)
Supplement: Supplementary file 1 — Additional file 1: Table SM.1. Expected (based on population) versus actual count of households receiving postcards. Fig. SM.1. “COVID Specific” postcard design. Fig. SM.2. “General Health” postcard design. Table SM.2. Descriptive characteristics of study sample by postcard type. Table SM.3. Likelihood of Agreeing with the statement: “Getting sick with COVID-19 can be serious.” Table SM.4. Likelihood of Agreeing with the statement: “I will probably get COVID-19.” Table SM.5. Likelihood of Agreeing with the statement: “The threat posed by COVID-19 is exaggerated by the Canadian federal government.” Table SM.6. Likelihood of Agreeing with the statement: “COVID-19 will NOT affect many Canadians.”. [file 12874_2022_1726_MOESM1_ESM.docx]

**Prospective Sampling Bias in COVID-19 Recruitment Methods: Experimental Evidence from a National Randomized Survey Testing Recruitment Materials**

**Supplementary Materials**

Eric B. Kennedy^1^ (corresponding, ebk@yorku.ca), Mia Charifson^2^, Megan Jehn^3^, Eric A. Jensen^4^, Jenna Vikse^5^

1. Disaster and Emergency Management, School of Administrative Studies, York University, Toronto, Canada

2. New York University School of Medicine, New York, United States

3. Global Health, School of Human Evolution and Social Change, Arizona State University, Tempe, United States

4. Department of Sociology, University of Warwick, Warwick, United Kingdom

5. Discourse, Science, Publics Lab, Department of Psychology, University of Guelph, Guelph, Canada

**Table SM.1. Expected (based on population) versus actual count of households receiving postcards**

Note the oversampling, as described in text, of NL, PE, YK, NT, and NU to account for small provinces/territories.

|  | **2019 Pop** | **Expected** | **Actual** | **Ratio of Actual Recruitment to Expected** |
| --- | --- | --- | --- | --- |
| **NL** | 521,922 | 2,137 | 5,836 | 2.73 |
| **PE** | 157,901 | 647 | 1,059 | 1.64 |
| **NS** | 976,768 | 3,999 | 4,109 | 1.03 |
| **NB** | 780,021 | 3,194 | 3,293 | 1.03 |
| **QC** | 8,522,800 | 34,896 | 36,109 | 1.03 |
| **ON** | 14,659,616 | 60,022 | 57,376 | 0.96 |
| **MB** | 1,373,859 | 5,625 | 5,765 | 1.02 |
| **SK** | 1,178,657 | 4,826 | 3,791 | 0.79 |
| **AB** | 4,395,586 | 17,997 | 16,735 | 0.93 |
| **BC** | 5,105,576 | 20,904 | 19,438 | 0.93 |
| **YK** | 41,022 | 168 | 492 | 2.93 |
| **NT** | 44,895 | 184 | 541 | 2.94 |
| **NU** | 38,873 | 159 | 214 | 1.34 |
| **Canada** | 37,797,496 |  |  |  |

**Figure SM.1. “COVID Specific” postcard design**

**Figure SM.2. “General Health” postcard design**

**Table SM.2. Descriptive characteristics of study sample by postcard type**

| **Characteristic** | **COVID-19 Specific**  **(n=1727)** | **General Health**  **(n=242)** | **Total**  **(n=1969)** |
| --- | --- | --- | --- |
| Age |  |  |  |
| Missing | 66 | 11 | 77 |
| Mean (SD) | 48.552 (16.525) | 52.463 (17.827) | 49.030 (16.733) |
| Gender |  |  |  |
| Missing | 2 | 1 | 3 |
| Female | 1036 (60.1%) | 127 (52.7%) | 1163 (59.2%) |
| Male | 689 (39.9%) | 114 (47.3%) | 803 (40.8%) |
| Education |  |  |  |
| Missing | 418 | 64 | 482 |
| Less than GED | 65 (5.1%) | 9 (5.1%) | 74 (5.1%) |
| GED | 261 (20.3%) | 42 (23.9%) | 303 (20.7%) |
| Trade certificate/diploma | 139 (10.8%) | 14 (8.0%) | 153 (10.5%) |
| College or other non-university certificate/diploma | 270 (21.0%) | 35 (19.9%) | 305 (20.9%) |
| University certificate/diploma below the bachelor’s level | 80 (6.2%) | 10 (5.7%) | 90 (6.2%) |
| Bachelor’s degree | 249 (19.4%) | 32 (18.2%) | 281 (19.2%) |
| University certificate/diploma degree above the bachelor’s level | 222 (17.3%) | 34 (19.3%) | 256 (17.5%) |
| Region |  |  |  |
| Missing | 50 | 5 | 55 |
| British Columbia | 171 (10.2%) | 37 (15.6%) | 208 (10.9%) |
| Maritimes | 162 (9.7%) | 23 (9.7%) | 185 (9.7%) |
| Ontario | 604 (36.0%) | 71 (30.0%) | 675 (35.3%) |
| Praries | 245 (14.6%) | 47 (19.8%) | 292 (15.3%) |
| Quebec | 480 (28.6%) | 54 (22.8%) | 534 (27.9%) |
| Territories | 15 (0.9%) | 5 (2.1%) | 20 (1.0%) |
| N-Miss | 50 | 5 | 55 |
| British Columbia | 171 (10.2%) | 37 (15.6%) | 208 (10.9%) |
| Maritimes | 162 (9.7%) | 23 (9.7%) | 185 (9.7%) |
| Race Identification |  |  |  |
| Missing | 54 | 9 | 63 |
| Non-Hispanic White | 1464 (87.5%) | 200 (85.8%) | 1664 (87.3%) |
| Non-Hispanic Asian | 101 (6.0%) | 17 (7.3%) | 118 (6.2%) |
| Multi-racial | 37 (2.2%) | 4 (1.7%) | 40 (2.1%) |
| Indigenous/Métis | 31 (1.9%) | 4 (1.7%) | 35 (1.8%) |
| Non-Hispanic Black | 16 (1.0%) | 3 (1.3%) | 20 (1.0%) |
| Hispanic origin | 15 (0.9%) | 3 (1.3%) | 18 (1.0%) |
| Non-Hispanic Arab | 9 (0.5%) | 2 (0.9%) | 11 (0.6%) |

**Table SM.3. Likelihood of Agreeing with the statement: “Getting sick with COVID-19 can be serious.”**

| **Variable** | **Odd’s Ratio** | **95% Confidence Interval** | **p-value** |
| --- | --- | --- | --- |
| Postcard Type |  |  |  |
| COVID-19 Specific | 1.00 | - | - |
| General Health | 0.714 | 0.522-0.976 | 0.035 |
| Age | 1.015 | 1.009-1.022 | <0.001 |
| Region |  |  |  |
| British Columbia | 1.00 | - | - |
| Maritimes | 1.267 | 0.792-2.025 | 0.323 |
| Ontario | 1.362 | 0.951-1.951 | 0.092 |
| Prairies | 1.494 | 0.991-2.253 | 0.055 |
| Quebec | 1.326 | 0.909-1.936 | 0.143 |
| Territories | 1.145 | 0.385-3.409 | 0.808 |
| Week | 1.035 | 0.916-1.17 | 0.580 |
| Gender |  |  |  |
| Female | 1.00 | - | - |
| Male | 0.726 | 0.586-0.899 | 0.003 |

**Table SM.4. Likelihood of Agreeing with the statement: “I will probably get COVID-19.”**

| **Variable** | **Odd’s Ratio** | **95% Confidence Interval** | **p-value** |
| --- | --- | --- | --- |
| Postcard Type |  |  |  |
| COVID-19 Specific | 1.00 | - | - |
| General Health | 0.739 | 0.561-0.972 | 0.031 |
| Age | 0.985 | 0.979-0.99 | <0.001 |
| Region |  |  |  |
| British Columbia | 1.00 | - | - |
| Maritimes | 0.832 | 0.556-1.244 | 0.370 |
| Ontario | 1.202 | 0.875-1.651 | 0.256 |
| Prairies | 1.109 | 0.78-1.577 | 0.566 |
| Quebec | 0.845 | 0.604-1.183 | 0.327 |
| Territories | 2.229 | 0.974-5.101 | 0.058 |
| Week | 0.937 | 0.845-1.038 | 0.212 |
| Gender |  |  |  |
| Female | 1.00 | - | - |
| Male | 1.137 | 0.948-1.363 | 0.166 |

**Table SM.5. Likelihood of Agreeing with the statement: “The threat posed by COVID-19 is exaggerated by the Canadian federal government.”**

| **Variable** | **Odd’s Ratio** | **95% Confidence Interval** | **p-value** |
| --- | --- | --- | --- |
| Postcard Type |  |  |  |
| COVID-19 Specific | 1.00 | - | - |
| General Health | 1.441 | 1.073-1.935 | 0.015 |
| Age | 0.983 | 0.978-0.989 | <0.001 |
| Region |  |  |  |
| British Columbia | 1.00 | - | - |
| Maritimes | 0.557 | 0.363-0.854 | 0.007 |
| Ontario | 0.695 | 0.502-0.962 | 0.028 |
| Prairies | 0.686 | 0.476-0.988 | 0.043 |
| Quebec | 0.636 | 0.451-0.898 | 0.010 |
| Territories | 0.464 | 0.177-1.216 | 0.118 |
| Week | 1.009 | 0.904-1.127 | 0.871 |
| Gender |  |  |  |
| Female | 1.00 | - | - |
| Male | 1.531 | 1.266-1.852 | <0.001 |

**Table SM.6. Likelihood of Agreeing with the statement: “COVID-19 will NOT affect many Canadians.”**

| **Variable** | **Odd’s Ratio** | **95% Confidence Interval** | **p-value** |
| --- | --- | --- | --- |
| Postcard Type |  |  |  |
| COVID-19 Specific | 1.00 | - | - |
| General Health | 1.213 | 0.918-1.604 | 0.175 |
| Age | 0.996 | 0.99-1.001 | 0.121 |
| Region |  |  |  |
| British Columbia | 1.00 | - | - |
| Maritimes | 0.787 | 0.519-1.193 | 0.259 |
| Ontario | 0.763 | 0.552-1.054 | 0.101 |
| Prairies | 1.015 | 0.709-1.453 | 0.934 |
| Quebec | 0.838 | 0.598-1.174 | 0.303 |
| Territories | 0.209 | 0.067-0.652 | 0.007 |
| Week | 1.035 | 0.932-1.15 | 0.516 |
| Gender |  |  |  |
| Female | 1.00 | - | - |
| Male | 1.393 | 1.156-1.679 | <0.001 |
